# Factors influencing pharmacists’ adoption of prescribing: qualitative application of the diffusion of innovations theory

**DOI:** 10.1186/1748-5908-8-109

**Published:** 2013-09-14

**Authors:** Mark J Makowsky, Lisa M Guirguis, Christine A Hughes, Cheryl A Sadowski, Nese Yuksel

**Affiliations:** 1 Faculty of Pharmacy and Pharmaceutical Sciences, 3-171 Edmonton Clinic Health Academy, University of Alberta, Edmonton, AB T6G 1C9, Canada

## Abstract

**Background:**

In 2007, Alberta became the first Canadian jurisdiction to grant pharmacists a wide range of prescribing privileges. Our objective was to understand what factors influence pharmacists’ adoption of prescribing using a model for the Diffusion of Innovations in healthcare services.

**Methods:**

Pharmacists participated in semi-structured telephone interviews to discuss their prescribing practices and explore the facilitators and barriers to implementation. Pharmacists working in community, hospital, PCN, or other settings were selected using a mix of random and purposive sampling. Two investigators independently analyzed each transcript using an Interpretive Description approach to identify themes. Analyses were informed by a model explaining the Diffusion of Innovations in health service organizations.

**Results:**

Thirty-eight participants were interviewed. Prescribing behaviours varied from non-adoption through to product, disease, and patient focused use of prescribing. Pharmacists’ adoption of prescribing was dependent on the innovation itself, adopter, system readiness, and communication and influence. Adopting pharmacists viewed prescribing as a legitimization of previous practice and advantageous to instrumental daily tasks. The complexity of knowledge required for prescribing increased respectively in product, disease and patient focused prescribing scenarios. Individual adopters had higher levels of self-efficacy toward prescribing skills. At a system level, pharmacists who were in practice settings that were patient focused were more likely to adopt advanced prescribing practices, over those in product-focused settings. All pharmacists stated that physician relationships impacted their prescribing behaviours and individual pharmacists’ decisions to apply for independent prescribing privileges.

**Conclusions:**

Diffusion of Innovations theory was helpful in understanding the multifaceted nature of pharmacists’ adoption of prescribing. The characteristics of the prescribing model itself which legitimized prior practices, the model of practice in a pharmacy setting, and relationships with physicians were prominent influences on pharmacists’ prescribing behaviours.

## Background

Healthcare systems around the world are implementing strategies such as expanded scopes of practice for healthcare professionals in order to improve primary health care delivery. One example is the expanded scope of pharmacy practice where pharmacists have been granted prescribing privileges to reflect the greater emphasis on a pharmacist’s role in promoting safe and effective medication use [1]. Prescribing activities range from extending refills or adapting a prescription to prescribing as part of a collaborative practice environment.

In 2007 Alberta was the first jurisdiction in Canada to have a full range of prescribing privileges [2,3]. Under these new standards, three types of pharmacist prescribing were defined: adapting a prescription, prescribing in an emergency, and prescribing at initial access or to manage ongoing therapy (i.e., additional prescribing authorization, APA). To obtain APA, pharmacists undergo a standardized peer evaluation of their education and training, experience and practice, and collaborative working relationships [4].

In 2002, the United Kingdom (UK) approved regulations which allowed supplemental prescribing (i.e., a form of dependent prescribing) allowing pharmacists to prescribe medications listed in a patient-specific clinical management plan agreed upon by the patient and an independent (i.e., autonomous), prescriber, typically a physician [5]. In 2006, the UK replaced supplemental prescribing with independent prescribing which requires pharmacists to demonstrate satisfactory prescribing skills through a training program. Similarly, several states in the United States (US) have legislation that supports dependent prescribing (i.e., prescribing delegated by another prescriber or protocol) as part of collaborative drug therapy management [6].

Prescribing, particularly initiating therapy or monitoring ongoing therapy, is a nascent expansion to the pharmacists’ scope of practice and Diffusion of Innovations theory can be used to understand this evolution. In essence, this theory states that, adopters (i.e., pharmacists) make choices on whether to embrace an innovation (i.e., prescribing) by examining the uncertain benefits and risks of this new innovation [7]. Diffusion of Innovations theory has been widely used in many disciplines to explore innovation in health care [8] as well as pharmacy practice [9,10]. Notably, Greenhalgh and colleagues have developed a conceptual model to describe the key determinants of diffusion, dissemination, and implementation of innovation in health service organizations [11].

The Greenhalgh model includes eight characteristics [11]. First, innovations likely to be adopted are clearly superior than the idea that it supersedes [7], are consistent with adopters’ values norms and perceived needs [7], are perceived as simple to use [7], can be experimented with on a limited basis [7], carry a low risk [11], and are relevant to the adopters’ work and improve task performance [11]. Second, adopter characteristics associated with the propensity to trial and use innovations include tolerance of ambiguity, motivation, values, and learning style [11]. Third, communication and influence activities lie on a continuum anchored by informal diffusion on one end and formal dissemination on the other. Fourth, system antecedents for innovation include the size or maturity of an organization, the absorptive capacity for new knowledge, and receptivity for change [11]. Next, system readiness for intervention relates to the organization’s ability to adopt a particular innovation. For example, pharmacists’ adoption may be facilitated by tension for change, support and advocacy by others in the health care system, and dedicated time and resources. Subsequently, the outer context is the external influences (e.g., political directives) that impact a decision to adopt, implement, and sustain an innovation. The seventh characteristic suggests that successful implementation can be facilitated by factors such as devolved decision-making and management support [11]. Finally, linkages such as those between innovators and potential users during the development stage may lead to more successful adoption [11].

Alberta offers a unique situation to study prescribing in pharmacy practice. Since there has been no formal assessment of the extent of adoption of prescribing by pharmacists in the province, the objective of this study was to understand what factors influenced pharmacists’ adoption of prescribing using the Greenhalgh model for the Diffusion of Innovations in health service organizations.

## Methods

This qualitative research was the first part of a three-phase project in which our overall goal was to characterize pharmacist prescribing practices in a larger sample [12]. In this first phase, qualitative methods were used to focus on “why” pharmacists are integrating prescribing to differing degrees. An Interpretive Description qualitative philosophy was used for the research design and analysis as it recognizes professional knowledge and the applied nature of addressing practice issues in health care [13]. This research was approved by the Health Research Ethics Board, University of Alberta, Canada.

### Study sample selection and sample size justification

Although the pharmacy work environment plays a prominent role in pharmacy practice [14], adoption occurs at the pharmacist level as prescribing is subject to an individual pharmacists’ professional discretion. We therefore purposefully and randomly selected pharmacists for the interviews using a variety of criteria: pharmacists prescribing with different practices, practice type, worksite, and workplace setting. We selected pharmacists who did not prescribe, pharmacists who were adapting prescriptions and/or prescribing in an emergency, and pharmacists with additional prescribing authorization. Pharmacists were recruited from independent and chain community pharmacies, hospitals, long-term care, and primary care networks [(PCNs) where pharmacists are situated in community clinics with interprofessional teams]. Alberta has a large and dispersed geographic area and approximately 3.8 million people live in the province. Pharmacists were selected from rural (i.e., village or town with a population < 10,000 citizens), small urban (i.e., greater than 10,000 citizens and smaller than large urban) and large urban (i.e., greater than 700,000 citizens) to reflect the population distribution (approximately 25%, 15%, and 60% respectively). No precise sample size was set *a priori* and sampling new sources occurred until redundancy when themes were repeated; all while acknowledging the potential presence of outliers.

### Methods and procedures for data collection

A list of all registered pharmacists who consented to using their information for research purposes was accessed from the Alberta College of Pharmacists (ACP). The listings were divided according to location (rural, small urban, and large urban) and then randomly assigned numbers. Potential participants were randomly selected and contacted by phone. Those who showed interest in participating were faxed or emailed an information sheet and consent form. A telephone or in-person interview was scheduled according to the participant’s availability stated on the signed consent form. Two phone calls were placed to each pharmacist before they were considered a non-responder. After responders were interviewed, further areas of participant need were identified. Pharmacists were then selected and purposefully contacted.

Thirty to forty-five minute semi-structured interviews, utilizing closed and open-ended questions, were conducted with pharmacists from August to November 2010. All interviews, except for one, were conducted over the telephone. Interviews were conducted by a trained research assistant and were digitally recorded and transcribed verbatim. Participants were informed of the audio recording on the written consent form as well as at the beginning of the interview. All identifying information was removed at the time of transcription. If elements of the transcript were not clear, pharmacists were contacted for clarification.

The interview questions flowed from the Diffusion of Innovations model and from prior literature [15,16]. We used a semi-structured qualitative interview guide to allow for increased flexibility and freedom when exploring the prescribing practices. Pharmacists initially completed a brief questionnaire on demographics, practice descriptors, experience, and education level. Pharmacists were asked to describe the last time they prescribed, types of prescribing they were currently using, their opinions about prescribing, their perceptions on the impact of prescribing on practice, barriers and facilitators, and their ideal prescribing situation.

### Analysis

Interpretive Description organizes the analysis into four stages including: comprehending the data, synthesizing to merge instances to understand typical patterns and outliers, theorizing to explain data synthesis, and re-contextualizing the findings to apply to other settings [13]. Transcripts were first read for understanding to describe each case and to establish an initial coding scheme. Each transcript was read by a minimum of two investigators, notes were taken and differences resolved through discussion. The Diffusion of Innovations model for health services organizations was the basis of the coding scheme [11]. To understand pharmacists’ adoption of prescribing, we focused on: the innovation of prescribing itself, the pharmacists as adopter, system readiness of prescribing, communication and influence and the outer context as pharmacists could reliably report on these constructs. We chose not to explore system antecedents for change (e.g., organizational vision), the implementation process (e.g., manager approaches), and linkages during the design or implementation stage as they would require an organizational lens on data collection and analysis.

Next, investigators independently conducted line-by-line coding. Data was coded into nVIVO 9. Each item of importance was coded accordingly and then reanalyzed at an individual level. Using constant comparison, we compared interview data with other interviews, theory, and literature. Notable barriers, facilitators, prescribing trends, and new codes developed and evolved through this analysis. Pharmacists were categorized as prescribers if they adopted prescribing as per the Alberta College of Pharmacists definition (i.e., adapt a prescription, prescribe in an emergency, and prescribe at initial access or to manage ongoing therapy). If pharmacists practiced in multiple sites, adoption of prescribing was based on the practice site with the most patient care activities (i.e., patient assessment and education).

## Results

In total, 399 pharmacists were contacted and 38 pharmacists were interviewed (Table 1) with representation from rural and urban centres as well as a variety of prescribing practices and pharmacy locales. Interviews ranged in duration from 18 to 51 minutes with an average of 27 minutes. There was an over representation of pharmacists with APA (i.e., 34% in our sample compared to less than 1% of pharmacists in Alberta with APA at that time) to allow for exploration of prescribing in this group of early adopters. The majority of pharmacists were community staff pharmacists. There was also representation from pharmacists in hospitals, speciality clinics, long term care, and PCNs, as well as owners, managers, practice leaders, and consultants.

**Table 1:** Participant characteristics

	***N = 38 *(%)**	
*Gender (male)*	14 (36.8%)	
*Additional Prescribing Authorization (Yes)*	13 (34.2%)	
*Years in Practice*		
1-5	6 (15.8%)	
6-10	5 (13.2%)	
11-15	3 (7.9%)	
16-20	7 (18.4%)	
21-25	3 (7.9%)	
26-30	6 (15.8%)	
30+	8 (21.1%)	
*Employer or Employee Status*		
Owner or Director	5 (13.2%)	
Clinical Practice Leader	3 (7.9%)	
Manager or Designate	5 (13.2%)	
Staff Pharmacist	22 (57.9%)	
Other	3 (7.9%)	
Practice Site 1*	*Site 1*	*Site 2*
Community: Chain or Franchise	11 (28.9%)	4 (10.5%)
Community: Independent	7 (18.4%)	1 (2.6%)
Hospital in-patient	5 (13.2%)	5 (13.2%)
PCN**	8 (21.1%)	1 (2.6%)
Speciality Clinic^†^	2 (5.3%)	1 (2.6%)
Other^‡^	5 (13.2%)	2 (5. 3%)
*Pharmacy Setting*		
Rural	11 (28.9%)	
Edmonton	13 (34.2%)	
Calgary	5 (13.2%)	
Urban Other	9 (23.7%)	
Work Status:		
Full time	32 (84.2%)	
Part time	5 (13.2%)	
Parental Leave	1 (2.6%)	

*If more than one practice, practice 1 is the site with the most patient centered care.

**Primary Care Network=Pharmacists work in a physician’s office as in an interprofessional team.

^†^Site 1&2== anticoagulation management services.

^‡^Site 1=3 long term care, 1 contract; Site 2=1 long term care, 1 hospital outpatient.

Using the Alberta College of Pharmacists definition, 29 out of 38 pharmacists were prescribing. The remaining nine pharmacists performed activities similar to prescribing (i.e., physician co-signed or pharmacist recommended therapy with a delegated protocol) or functioned as part of an integrated team where medication recommendations were incorporated into the physicians’ prescribing practices.

**Figure 1:**
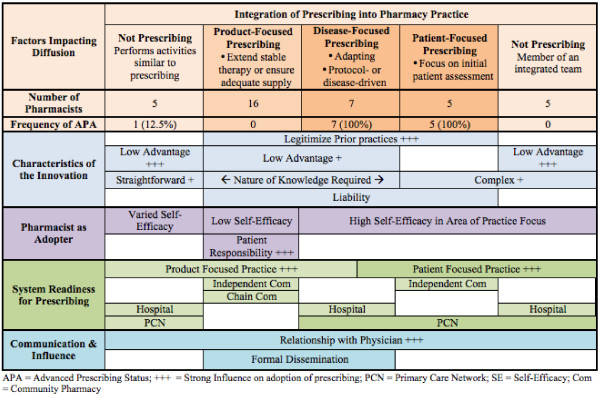
Key factors that influenced pharmacist prescribing.

In order to understand influences on prescribing, we characterized pharmacists prescribing as product, disease and patient focused (Figure 1). In product prescribing, pharmacists were motivated to prescribe if there was a physical lack of the medication (e.g., manufacturer shortage) or if the patient required a refill of ongoing medication therapy. Pharmacists in the disease focused group prescribed as per practice guidelines or protocol specific to their practice and tended to focus in one specialized disease area. Finally, pharmacists who were patient focused prescribed to initiate therapy based on their patient assessment and when monitoring patient outcomes.

### What factors influenced pharmacists’ adoption of prescribing?

Influences on pharmacist prescribing were grouped into five categories based on Greenhalgh’s conceptual model for Diffusion of Innovations [11] (Figure 1): 1) the innovation, 2) the pharmacist as adopter, 3) system readiness for prescribing 4) communication and influence, and 5) outer context.

### Legitimization of previous practices

Pharmacists identified a need for prescribing whether it was switching drugs because of a manufacturer’s shortage, adapting the dose, or stepping in when physicians were not available. In the past they had found ways to meet this societal need by providing additional medications until a physician’s prescription was obtained. Some pharmacists described prescribing as legitimizing the practice of loaning a patient a few tablets in community or making therapy recommendations in the hospital (Figure 1). This theme was present in all pharmacists who were prescribing, but was articulated most clearly by community pharmacists (Table 2).

**Table 2:** Selected quotes by thematic categories

**Category**	**Theme with selected quotes**
Characteristics of the innovation of prescribing	Legitimization of Previous Practices:
	*Before we had prescribing, we did continuity as well. We just provided the medication and got the ok from the doctor the next day – kind of thing. It just changed the procedure; it hasn’t really changed what we do‘ (P14, male, rural independent community pharmacist).*
	Relative Advantage:
	*‘I’m traditionally a hospital pharmacist and I couldn’t be doing community if all of these changes hadn’t happened to access prescribing, additional, expanded scope of practice’ (R 31, female, owner community pharmacy, PCN pharmacist).*
	Nature of Knowledge Required:
	*‘I will give you a classic example. Friday night a young lady comes in looking for birth control; she was out of refills and has an appointment next month…. There are very few adverse effects or harm. There is potential for harm to come if you don’t give the medication. So you do the adaptation’ (R19, male, rural Community Pharmacist).*
	Liability:
	*‘There was far more risk under the old system because you were not legally allowed to do what you were doing most of the time’ (P19 male, rural community chain manager, No APA).*
	*‘initial access prescribing, … That’s a road we’ve got to be really careful going down because it’s full of goblins. It’s full of risks, it’s full of potholes’ (P26, male, urban hospital pharmacist).*
	Workload and Trialability:
	*‘Well, it is an onerous process. It is not easy to do I am finding…. all of the steps and then my colleague sent in his application and got rejected so I am starting to think oh man, I have got to work hard at that’ (R14, male, owner rural community pharmacy).*
The pharmacist as adopter	Knowledge, Skills and Confidence:
	*‘I’m sure that a lot of the younger people should be doing this, if they’re not. And us old guys will kind of fade out anyway’ (P29, male, urban PCN pharmacist).*
	Beliefs and Emotions about Prescribing:
	*‘…feels, let’s see, I think it’s a little scarier. For me because patients expect more of you and they know your role is this and such and such, and I didn’t go to school learning that and I’ve been out for a while’ (P33, female, urban community chain pharmacist).*
	Motivation:
	*‘I’m not as gung ho as I probably should be and I guess it’s basically kinda almost fear’ (P11, female, urban community chain pharmacist).*
	*‘I’m certainly not a pharmacist who will sit on a stool and work till midnight somewhere for extremely good money (laughter). I’d rather put all my money on the line and make a difference’ (P13, APA, male, rural independent pharmacy owner).*
	Beliefs about Patient Responsibility:
	*‘But I guess we have to teach people what the importance is of, the doctor prescribed them a year’s worth, it now says zero, I think they want to see you again’ (P30, male, rural community independent pharmacist).*
System readiness for prescribing	Innovation-system fit:
	*‘It is too – it’s a grocery store. It needs to be in a clinical setting….’ (P10, female, urban community chain pharmacist).*
	*‘I just think of how just our practice environment currently is set up. Just time*
	*challenges, also just shortages of pharmacists as well, and also more training up of technicians as well, to take on more responsibility. Also, just acceptance by other health care professionals. Patients – educating them as well. So I think there will be challenges but it will be a really great opportunity too’ (P 37, female, urban community chain pharmacist).*
	*‘And then of course now, they are saying “why are you not prescribing?” and I say when you are truly in a collaborative practice like I am, we discuss prescriptions, there is a conversation back and forth between the nurse practitioner/doctor/doctors and it becomes irrelevant who puts it into the electronic health record generates it and signs it’ (P20, female, urban primary care consultant).*
	*‘For a primary care network pharmacist it just makes sense and it fits into our practice. I can see some of the concerns from both sides from the community pharmacists and their sort of … feeling like they are on an island and they don’t have enough information to make this decisions to prescribe’ (P12, female, urban PCN manager).*
	*‘You can spend more time with the patient and make the best decision for them [in the PCN]. Whereas the community is a very face paced setting. Most patients don’t have an extra 10 minutes to sit down with you because they’re on their way home from grocery shopping and have to rush to pick up their kid from soccer, that type of thing. Whereas, in the primary care network, you’re sitting in an office and they’ve committed that time to you. It’s important. The patient sees it as important. And I don’t know, it could still work in the community, but I think it would be more difficult’ (P27, male, urban PCN, chain community pharmacist).*
	Support:
	*‘And they actually made me a prescription pad, that’s kind of cool (laughter)’ Pharmacist 34 (APA, female, urban PCN practice).*
Communication and influence	Relationship with Physicians:
	*‘Contact the doctor first and see if we can get [a refill]’ (P37, female, urban community chain pharmacist); and prescribe ‘as a last resort if the physician was not available’ (P24, male, urban PCN, community pharmacy).*
	*‘I feel the independence and the freedom is second to none and I am not at the mercy of a physician that I can rarely get in touch with and have to chase down and stuff. It’s – if I know what they need and I am confident and sure and they are willing to let me help them, that I can do it and I don’t need to rely on someone else to do it’ (R4, Community, urban LTC care pharmacist, APA).*
	*‘I have had one doctor – not a local doctor– who told his patient that if she ever had a pharmacist extend her prescription again that she would not be his patient anymore, but I haven’t…other than that, I haven’t had any real negative reactions’ (P14, male, rural independent community pharmacist).*
	Formal dissemination:
	*‘I would think just the constant reminders from the college about “hey, don’t forget, you guys can do this and just make sure you are embracing that”’ (P24, male, urban PCN pharmacist).*
	Informal diffusion: n/a
Outer context	*‘…without the funding it’s not something we’ve done a whole lot with’ (R9, APA, female, rural Community/PCN pharmacist).*

#### Relative advantage

Pharmacists who had adopted prescribing practices named many relative advantages of prescribing in their practice. Prescribing had increased their sense of professionalism, the image of the professional healthcare provider and their own job satisfaction and happiness. Prescribing was convenient for the physician and patient in maintaining continuity of prescription medications. A pharmacist with APA felt prescribing *“even potentially made the docs a little bit more appreciative of what I can do” (P34, APA, female, urban PCN practice).* The community pharmacists with APA felt that the ability to prescribe had increased their job satisfaction (Table 2).

At the time of data collection the government did not reimburse pharmacists’ prescribing services. Still, adapting prescriptions made good business sense since the pharmacy received compensation for the prescription and prescribing generated customer loyalty. Pharmacists related that some customers did not appreciate paying the professional fee for a small number of pills (e.g., a week supply) resulting in some pharmacists prescribing for longer time periods.

Pharmacists who felt there were few advantages to prescribing were less likely to prescribe or obtain APA (Figure 1). Pharmacists did not directly receive monetary benefits and retail pharmacies did not require prescribing as a condition of employment. One pharmacist, *(P 20, female, urban primary care consultant)* did not obtain APA as she felt she would have less time for working directly with patients; she “*would be stuck with more refills then I am already”* which required her to prescribe for patients’ refill requests based on a review of patients’ charts.

#### Nature of knowledge required

The knowledge required for prescribing can be complex, require clinical judgement, and rely on multiple sources of information. For example, pharmacists recognized that prescribing often required a diagnosis available from a physician. Pharmacists were most comfortable prescribing for stable patients on chronic medications who were well known to the pharmacist. They used terms such as something minor, straightforward, tweaking, or non-invasive situations to describe ideal prescribing situations (Table 2).

In contrast, pharmacists were less likely to prescribe in more complex situations where the patient was on multiple medications, was not stable, had unclear diagnosis, or did not fit into typical clinical guidelines (Figure 1). Half of pharmacists with APA (seven out of 13) were most often prescribing in areas with explicit protocols including appropriate dose ranges such as anticoagulation therapy.

#### Liability

Pharmacists related concerns over the risk or liability associated with prescribing (Figure 1). Pharmacists managed this liability by reducing the amount they prescribed, taking care to document and taking additional time to review clinical information. Some pharmacists identified high-risk medications that they would not consider prescribing, such as anticoagulation therapy, antipsychotics, and sedatives. Pharmacists’ concerns about liability generally lessened with experience prescribing. Notably one pharmacist who routinely adapted prescriptions, felt prescribing reduced risk (Table 2).

Overall, most pharmacists believed that APA had increased risk (Table 2).

In contrast, Pharmacist 12 (APA, male, rural independent pharmacist) felt that “*good sound science*” would protect him from liability and went on to say that he would prescribe drugs that some pharmacists may consider as higher risk (e.g., prednisone).

#### Workload and trialability

Pharmacists felt that prescribing added to their workload mainly due to requirements for documentation and prompt communication with physician, though pharmacists reported this became easier over time. APA was not subject to trialability as it required substantial paperwork, commitment to undergo peer review and could not be implemented on a limited basis. Pharmacists felt overwhelmed by the APA application process, particularly when colleagues were denied (Table 2).

Trialability of APA was also limited by the role of physician as gatekeeper as part of the APA application process. Often finding a prescriber to write a letter of support for an APA application was the rate-limiting step. Pharmacist 7 (male, rural chain manager) found that physicians opinions about other pharmacist colleagues prevented them from supporting his APA application despite close personal and working relationships. Workload and Trialability were not placed in Figure 1 as these factors appeared to have limited impact on adoption of prescribing.

### The pharmacist as adopter

#### Knowledge, skills and confidence

Pharmacists identified the need for enhanced knowledge, skills, and self-efficacy to provide higher levels of patient care including prescribing. Pharmacists with product focused prescribing were more likely describe low self-efficacy toward prescribing (Figure 1). Most pharmacists were under the misconception that a clinical focus (e.g., diabetes, asthma, anticoagulation) was required to obtain APA. Some pharmacists felt that they were too advanced in their career to adopt prescribing practices (Table 2).

#### Beliefs and emotions about prescribing

Pharmacists shared the belief that a pharmacist prescriber takes additional responsibility for the medication and shared emotions about prescribing from happiness at the new opportunity to fear and apprehension about the new responsibility (Table 2).

#### Motivation

Pharmacists with product focused prescribing reported lower motivation than pharmacists who had adopted disease or patient focused prescribing. Personal motivation toward pharmacists’ use of prescribing was illustrated in two contrasting quotes (Table 2).

#### Beliefs about patient responsibility

Some community pharmacists did not wish to prescribe in place of regular physician visits and felt it was the patient’s responsibility to obtain timely refills (Figure 1)*.* For example, Pharmacist 27 *(female, PCN, community chain pharmacist) “informs the patient that it’s a one-time thing”* to avoid being caught in the middle between the patient and the physician. Pharmacists felt uncomfortable in helping the patient avoid routine physician visits (Table 2).

### System readiness for prescribing

#### Innovation-system fit

Pharmacists with product, disease or patient focused practices adopted prescribing in a manner reflecting those practice styles (Figure 1). Pharmacists with all types of prescribing practice stressed the important of innovation-system fit though its nature differed among practice site. Commonly, pharmacists felt the community pharmacy environment was not conducive to disease or patient focused prescribing due to a lack of time for patient assessment, collaboration, clinical information, and general environment; thus the majority of community pharmacists prescribed to ensure continuity of stable therapy (i.e., product focused prescribing in Figure 1) (Table 2).

Hospital pharmacists were split on the usefulness of prescribing. Some adopted disease focused prescribing while others with clinical practices did not adopt prescribing (Figure 1). While the hospital environment provided access to patients, complete histories, and other healthcare providers, some institutional policies that were in place prior to 2007 did not allow pharmacists to prescribe. Even so, some pharmacists performed prescribing-like activities where they made therapeutic interchanges based on formularies, adjusted medication levels based on guidelines and lab values, or wrote medication orders that were co-signed by a physician. In the hospital setting or ambulatory clinic setting, some pharmacists did not feel the need to prescribe themselves as they were integrated into interprofessional teams where they had sufficient input on prescribing decisions (Table 2).

Many respondents in both community and hospital practice regarded a PCN as the ideal environment as it afforded time for patient care, access to medical records, and face-to-face interactions with other health care professionals (Table 2).

Several PCN pharmacists, particularly those without APA, did not prescribe for continuity of therapy as they did not dispense medications and therefore did not have an original prescription to adapt (i.e., a legal requirement of adapting a prescription). As a result, PCN pharmacists adopted disease and patient focused prescribing (Figure 1).

The impact of innovation-system fit was most markedly observed with eight pharmacists who worked in more than one practice setting. One pharmacist worked in three outpatient clinic settings with similar practice activities and subsequently had similar clinical prescribing activities across these sites. The remaining seven pharmacists varied their prescribing activities to match the practice setting with product-focused prescribing at one site and disease or patient focused at the other. Product-focused prescribing was associated with community practice for the majority pharmacists (i.e., six of seven pharmacists who worked in more than one setting). For example, several pharmacists prescribed with a product-focused focus in community and a disease or patient focus in their PCN practice (Table 2).

#### Support

Pharmacists were more likely to prescribe when they had more support than opposition. Pharmacists who worked in healthcare teams within PCNs or institutions felt that team member support was crucial to their success. At times there was tangible evidence of this support (Table 2). Pharmacists recognized the need and expressed desire for support by management to move forward with prescribing. When asked about resources needed to prescribe, pharmacists most commonly mentioned time and staffing, access to lab values through the provincial electronic health record, existing clinical programs, and the need for access to medical journals.

### Communication and influence

#### Relationship with physicians

Pharmacists described how the nature and extent of their relationship with physicians had substantial influence pharmacist prescribing. The physician relationship was often a primary consideration for pharmacists when deciding whether to prescribe for a patient (Figure 1). Many pharmacists expressed reluctance at prescribing when they believed the physician was not supportive of pharmacist prescribing and varied their prescribing practices based on the patient's primary physician. This was particularly the case in community pharmacy where many pharmacists preferred to contact the physician to obtain authorization for refills in place of prescribing (Table 2). In contrast, one pharmacist who worked part-time in a community chain felt APA meant she was “not at the mercy of a physician” and was not concerned about the physician's opinion (Table 2). Overall, pharmacists described a pattern where the majority of physicians supported or encouraged their prescribing with a few prominent negative experiences where a physician was not supportive (Table 2).

#### Formal dissemination

The Alberta College of Pharmacists was recognized for responding to a policy window when they created the legislation for prescribing. The Alberta College of Pharmacists was perceived as the primary source of information and encouragement to prescribe (Table 2).

In community pharmacy practice, pharmacists with product-focused prescribing practices regularly relied on forms and protocols developed by their corporate offices to help guide and support their prescribing practices.

#### Informal diffusion

Pharmacists who networked with other colleagues, other health care providers, or research networks had more active prescribing practices. One pharmacist had identified a pharmacist mentor while four other pharmacists felt their access to physicians was crucial in learning how to prescribe. Physician offices also referred patients to the pharmacy to obtain refills when a physician’s appointment was not available. Similarly, patients who valued pharmacist prescribing referred others.

### Outer context

There were few references to elements of the outer context such as the social political climate and environment; thus it was not included in Figure 1 as it was not a strong influence. Pharmacists referred to a lack of reimbursement for their services, particularly prescribing by government or drug plans as a primary barrier to practice in our provincially funded healthcare system (Table 2).

## Discussion

Pharmacist prescribing provided convenience to patients and physicians as well as improved the professional image of pharmacists. A constellation of factors influenced pharmacist prescribing; however, the most prominent were the characteristics of the prescribing model itself that legitimized prior practices, pharmacy site’s model of practice (i.e., innovation system fit), and physician relationships that impacted prescribing behaviours and individual pharmacists decisions to apply for independent prescribing privileges (i.e., APA). Other less prominent factors included prescribing advantages, pharmacist self-efficacy, patient relationships, and formal dissemination.

One of our three key themes was that prescribing facilitated pharmacists’ completion of tasks they were performing prior to the introduction of legislation in 2007. This phenomenon has been labelled compatibility in the classic Rogers theory [7] and system-innovation fit in Greenhalgh’s model of innovation health services organization [11]. Pharmacists who were providing high levels of patient care and making therapeutic recommendations to physicians were using APA to implement care plans independently. Community pharmacists valued the ability to issue a prescription for continuity of care as this legitimized the prior practice of providing medication without a prescription until a patient could see a prescriber (i.e., “legalized loaning”). In essence, practice had not changed; prescribing altered how these pills were accounted for in the software system and the physician was informed after the pills were released to the patient. Similarly, innovation compatibility has been shown to enhance pharmacists’ adoption of written medication information [17], and pharmacists’ provision of immunization services [18].

A second key finding was practice setting strongly influenced the adoption of prescribing. This replicates earlier findings that pharmacists practice behaviours in Michigan predicted uptake of an advanced service for patients with hypertension [19]. Pharmacists expressed a concern that the community pharmacy environment was not suitable with its isolation from other healthcare providers, lack of patient information, fast pace, and focus on technical task of accurately preparing medications. Furthermore, community pharmacy space may not invite professional healthcare interactions [20]. Pharmacists perceived hospitals and primary care clinics as more conducive environments to prescribing because they had more team based opportunities. Despite this, pharmacists who worked in PCNs could not prescribe without APA as they did not dispense the physical prescription to adapt. Some hospital pharmacists found prescribing offered no distinct advantage as were embedded within teams and already contributed to prescribing decisions.

Third, relationships with physicians impacted prescribing behaviours. Pharmacists cited examples of physicians who asserted their authority to maintain the status quo. In contrast, there were also examples of physicians who mentored pharmacists, wrote letters of support for APA applications, and referred patients for pharmacists prescribing. Either way, physicians maintained influence over pharmacist prescribing. Alberta pharmacists appear to prescribe in such a way to avert physician conflict. Pharmacists were hesitant to prescribe if they believed the physician was unsupportive and limited their focus to certain disease areas and medications [21]. When establishing collaborative relationships, pharmacists have the responsibility to initiate physician relationships with face-to-face interactions and demonstrate high quality contributions to care [15,22,23]. In the case of community pharmacy, this power asymmetry is apparent as the level collaboration is often dependent on the physician’s endorsement of the pharmacist [24].

As predicted by role theory, pharmacists who did not believe prescribing was part of their professional role were less likely to prescribe [25]. The decision to prescribe resides with the pharmacist as Alberta’s regulation are enabling and do not compel individual pharmacists to prescribe [26]. A few community pharmacists believed patients should ensure regular physician’s appointments and it was not the pharmacist’s responsibility to prescribe for continuity of therapy. Pharmacists’ reluctance may result from the desire to avoid a disruption of the patient-physician relationship [27].

### Comparison to pharmacist experiences with prescribing in other jurisdictions

Our findings are highly consistent with studies of pharmacists taking on new prescribing practices in the other jurisdictions including the UK and US. As with our own findings, supplementary prescribers in the UK were more likely to prescribe if they were “legitimizing” prior practices in the context of existing teams or relationships [28,29]. In the UK, pharmacists raised concerns over documentation and workload. Specifically, pharmacist felt the clinical management plan written between the physician and pharmacist in UK supplemental was an excessive bureaucratic burden [30]. Thus, UK pharmacists felt supplementary prescribing was a “stepping stone” to independent prescribing which has fewer administrative burdens [30]. UK pharmacists who did not have a good system-fit felt they were going down a “dark alley” and did not implement prescribing [28]. Similarly, pharmacists in the UK felt the added liability particularly when prescribing “riskier” medications and subsequently stressed the importance of staying within their area of competence [29]. In the UK, pharmacists were positive about prescribing and felt concerns with the training model, pharmacists’ competence, and implementation would be resolved over time [31].

With respect to pharmacist – physician relationships, in the UK, Weiss concluded that the current prescribing models have not challenged the physician’s dominance [21]. UK physicians have expressed a preference for supplementary over independent prescribing where they can assert control over pharmacists prescribing [30,32,33]. Blenkinsopp found that UK physicians maintained control over prescribing when working with independent prescribers by delegating “some routine work that did not require diagnosis and only limited decision making” [34]. Pharmacist do not diagnose except in the case of minor ailments managed by non-prescription therapy, therapies which physicians have clearly indicated as the sole domain for pharmacists [35].

### Limitations

Response bias would suggest that pharmacists who responded were likely to prescribe. Additional limitations include that our methodology did not follow experiences over time and lacks the rapport offered by face-to-face interviews. We experienced challenges in recruitment, mostly as a result of only having home phone numbers for potential pharmacist participants. Perhaps due to the nature of the questions or the perspective of pharmacists, the outer context was the area with the fewest references. A systems approach would be useful to fully appreciate pharmacists’ use of prescribing.

### Implications for research

The Diffusion of Innovations model was useful in explaining pharmacists’ adoption of prescribing. Research on pharmacist prescribing should focus beyond the individual pharmacist and expand to include the pharmacy practice model and its business environment [14]. Our adaptation of Diffusion of Innovations theory for pharmacist prescribing requires further testing across different jurisdictions.

### Implications for practice

Practice should consider interventions at the health system level to examine demand for pharmacist prescribing, healthcare professionals’ perceptions, and patient expectations. Prescribing practices mirror the practice level of the site; thus in order to increase prescribing, one would have to enhance the practice level for the site. Pharmacists should consider specific actions to build trust and develop collaborative relationships with physicians in the context of pharmacist prescribing. Interventions aimed at increasing pharmacist prescribing should address these issues alongside training on knowledge and skills to ensure pharmacists develop strong self-efficacy toward prescribing.

## Conclusions

We found that Diffusion of Innovations theory was helpful in understanding the multifaceted nature of pharmacists’ adoption of prescribing. Based on our qualitative study, we found that pharmacists’ prescribing was characterized as product, disease and patient focused. The adoption of these prescribing activities were dependent on a constellation of factors, with the most prominent being the legitimization of prior behaviours, influence of the current practice setting, and the quality of pharmacist-physician relationship.

## Competing interests

The authors declare that they have no competing interests.

## Authors’ contributions

LMG, CAH, MJM, CAS, and NY participated in the design of the study, and analyses. MJM and LMG wrote the first draft of the manuscript, and all authors were involved in critically evaluating the manuscript. All authors read and approved the final manuscript.
